# Electric and Hydraulic Properties of Carbon Felt Immersed in Different Dielectric Liquids

**DOI:** 10.3390/ma11040650

**Published:** 2018-04-23

**Authors:** Alexey Kossenko, Svetlana Lugovskoy, Moshe Averbukh

**Affiliations:** 1 Department of Chemical Engineering, Ariel University, Ariel 40700, Israel; kossenkoa@ariel.ac.il (A.K.); svetlanalu@ariel.ac.il (S.L.); 2 Department of Electrical/Electronic Engineering, Ariel University, Ariel 40700, Israel

**Keywords:** carbon felt, dielectric liquids, electrical conductivity, hydrodynamic permeability

## Abstract

Electroconductive carbon felt (CF) material, having a permeable structure and significant electroconductive surface, is widely used for electrodes in numerous electrochemical applications such as redox flow batteries, fuel cells, electrochemical desalination apparatus, etc. The internal structure of CF is composed of different lengths of carbon filaments bonded together. This structure creates a large number of stochastically oriented and stochastically linked channels that have different lengths and cross sections. Therefore, the CF hydraulic permeability is similar to that of porous media and is determined by the internal empty volume and arrangement of carbon fibers. Its electroconductivity is ensured by the conductivity of the carbon filaments and by the electrical interconnections between fibers. Both of these properties (permeability and electrical conductivity) are extremely important for the efficient functioning of electrochemical devices. However, their influences counter each other during CF compressing. Increasing the stress on a felt element provides supplementary electrical contacts of carbon filaments, which lead to improved electrical conductivity. Thus, the active surface of the felt electrode is increased, which also boosts redox chemical reactions. On the other hand, compressed felt possesses reduced hydrodynamic permeability as a result of a diminished free volume of porous media and intrinsic channels. This causes increasing hydrodynamic expenditures of electrolyte pumping through electrodes and lessened cell (battery) efficiency. The designer of specific electrochemical systems has to take into account both of these properties when selecting the optimal construction for a cell. This article presents the results of measurements and novel approximating expressions of electrical and hydraulic characteristics of a CF during its compression. Since electrical conductivity plays a determining role in providing electrochemical reactions, it was measured in dry conditions and when the CF was immersed in several non-conductive liquids. The choice of such liquids prevented side effects of electrolyte ionic conductivity impact on electrical resistivity of the CF. This gave an opportunity to determine the influences of dielectric parameters of electrolytes to increase or decrease the density of interconnectivity of carbon fibers either between themselves or between them and electrodes. The experiments showed the influence of liquid permittivity on the conductivity of CF, probably by changing the density of fiber interconnections inside the felt.

## 1. Introduction

CF material is widely used for electrodes in different electrochemical applications [1,2,3,4]. Nowadays, it’s most noticeable utilization is in Vanadium Redox and other flow batteries due to its good electrical conductivity, hydraulic permeability, and durability in strong acid and alkali electrolytes [5,6,7,8,9,10,11].

As a material for electrodes, CF must possess good electrical conductivity and allow electrolyte flow through its framework. Its conductivity could be improved by felt compression due to the diminishing distance between carbon fibers and producing additional fiber interconnections. However, this automatically causes a decrease in empty volume and cross section of micro-channels connecting pores inside the felt framework. The result of this process is a decrease of felt permeability or an increase in hydraulic resistance, which in turn diminishes the flow velocity through the felt electrode and causes additional hydrodynamic losses.

Since for the optimal development of a specific electrochemical cell, one has to take into account both hydraulic and electric parameters, numerous scientific works have studied them experimentally and theoretically.

Various approaches have been applied in hydrodynamic characteristic studies. Significantly different approaches are found in the literature for describing the permeability of CF electrodes [12,13,14,15,16]. While [12,13] uses a simplified description of felt hydrodynamics with a Darsy approximation, [14,15,16] are based on a much more complicated theoretical representation of Kozeny–Carman equations with a modified Reynold’s number. 

In all cases estimation of average liquid velocity (*u*) having viscosity (*µ*) through porous with length (*L*) media under a pressure drop (∆*P*) is given by [16]:

(1) $$u=-\frac{k}{\mu }\frac{\Delta P}{L}$$

The expression of permeability coefficient (*k*) for CF, dependent on the fiber’s diameter (*d*) and the felt porosity (*λ*), is:

(2) $$k=\frac{d^{2}}{a\cdot K_{CK}}\cdot \frac{\lambda^{3}}{{\left(1-\lambda \right)}^{2}}$$

where a and *K_CK_* represent special constants that in some works are combined into a single coefficient (*C*). For example, in [17], the permeability coefficient has the following more common value:

(3) $$\frac{k}{d^{2}}=\frac{1}{C}\cdot \frac{\lambda^{n+1}}{{\left(1-\lambda \right)}^{n}}$$

where both *C* and *n* are to be obtained from experimental tests.

The hydrodynamic characteristics of CF were successfully investigated by complicated theoretical approaches [17,18,19]. In [17] the description of a fiber porous medium was made using the Voronoi Tessellation approximation, which improves the application of the Kozeny–Carman equation for hydraulic properties estimations of a felt with a significantly random structure. The method in [18] suggests a representation of a high porosity fibrous media by a bundle of relatively small repetitive unit cells randomly dispersed inside a felt. An assessment of liquid flow velocity in this work is based on the Voronoi Tessellation approximation. Theoretical results were obtained for one-, two- and three-dimensional (1D, 2D, and 3D) felt structures as well as for perpendicular and parallel flow directions. Corresponding with that of [17,18], the method in [19] of using a difference-fractal model for the permeability of viscous liquid flow through fibrous media was suggested. The results showed good agreement with the experimental data from different literature sources. Emphasizing the significant theoretical backgrounds and mathematical solutions of [17,18,19] that provide strict analytical description of porous fibrous media hydraulic permeability, it should be noted that the practical use of their results is problematic. This was supported by the fact that the obtained values of permeability coefficients versus porosity factor and radius of fibers are presented on a logarithmic scale. As a result, small inaccuracies in the initial conditions, which are difficult to determine precisely in practical use, could significantly change the calculated result of the permeability value, thus diminishing the efficiency of the method. Therefore, we decided to apply a simplified approach based on experimental investigation of a specific CF with a subsequent mathematical approximation of experimental results, which will be described in the following section. 

In contrast to hydraulic properties, electrical properties of CF were considerably less studied.

Historically, one of the first investigations of CF electrical resistivity used for heating elements is found in [20]. The following research work [21] describes the design of a new bipolar cell with a proton-exchange membrane where flat electrodes were made of flexible and loose carbon fiber bunches representing an analog of CF. Due to the need to ensure good electrochemical reactions on electrodes, tests of CF resistivity were conducted. For the first time, the results of CF area-specific resistance were presented as a function of squeezing pressure. It should be noted that average surface resistance (ASR) represents a parameter equal to a total resistance in the perpendicular-plane direction of CF electrodes divided by the value of its area. The decrease of the bulk resistance was observed as an exponential function, with the negative exponent value depending on the pressure applied to the CF electrode.

Important and original results of measured ASR for CF [22] were expressed as a function of a compression pressure. Different electrode–bipolar plate assemblies and adhesive conducting materials were investigated in this work.

The same (as in [21]) exponential diminishing of a resistance versus compression pressure was observed, however, with different coefficients of the exponential function, which depend on the type of construction and adhesive materials. However, the abovementioned references do not include an analytical expression of resistance versus felt porosity and specific resistivity of a carbon filament. This shortcoming is improved in [23,24,25]. Specific area electrical resistance ($\rho_{felt}$) [23] is approximated by electrical resistance of a carbon material ($\rho_{felt}$) comprising felt filaments and by a felt porosity as:

(4) $$\rho_{felt}=\frac{4}{\left(1-\lambda \right)}\rho_{carbon}$$

Equation (4) is in good agreement with the measurements results provided in [24]. ASR of CF electrodes (denoted as (*R_ASR_*) in [25]) for vanadium redox batteries was determined experimentally. Approximation of *R_ASR_* (mΩ·cm^2^) versus *V_f_* (fiber volume fraction, %, the opposite of the porosity *λ*, i.e., *V_f_*/100 = 1 − *λ*) was evaluated by following exponential function:

(5) $$R_{ASR}=144.456\cdot {\left(3-\frac{10}{V_{f}}\right)}^{-1.117}$$

Equation (5) is more accurate for describing electrodes’ resistance of specific vanadium redox batteries assumes only carbon content. Also, the important roles of a specific carbon conductivity value and interconnections density between carbon filaments inside a felt, were not taken into account. Furthermore, all experimental measurements were carried out in dry conditions only, i.e., in air. Thus, neglecting fibers interconnections in Equation (5) cannot precisely predict electrical conductivity of a CF being immersed in different electrolytes. However, real electrodes work in electrochemical cells filled with different electrolytes and our experiments showed the dependence of resistivity on the type of electrolyte. The resistivity or conductance of CF seems to be influenced by some liquids’ specific properties, conducive to the penetration of fluid between fibers, in this way decreasing the interconnection density and diminishing the felt conductivity or, conversely, causing the convergence of fibers and a decrease in resistance.

Because of the significantly stochastic framework of the felt structure, theoretical analysis as a rule requires many assumptions, making the real-world validity of its results questionable. 

Therefore, it was decided to conduct a parallel study of the electrical and hydraulic properties of the same CF. The results of the experimental research together with the analytical approximations are presented below.

## 2. Methodology of Experimental Research

The experimental research main objective was the investigation and determination of hydraulic and electrical properties of CF in a wide range of external conditions.

### 2.1. Characteristics of CF

**Figure 1:**
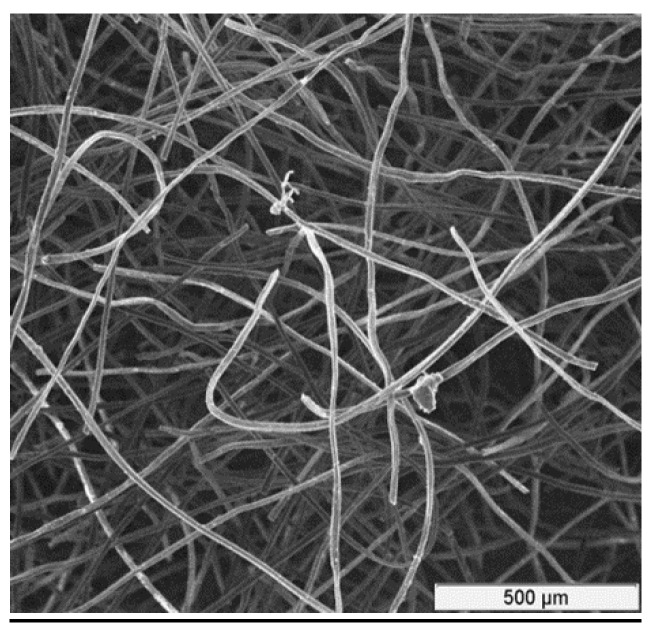
A magnified picture of the CF structure.

The CF for experiments was a rayon-based material made from high-quality carbon fibers. It has a porous structure with large surface area. Rayon-based CF from “Mersen Graphite”, Greenville, MI, USA [26] was used for the tests. Its structure is shown in Figure 1. Additional characteristics of this type of CF are shown in Table 1 ([27]).

**Figure 2:**
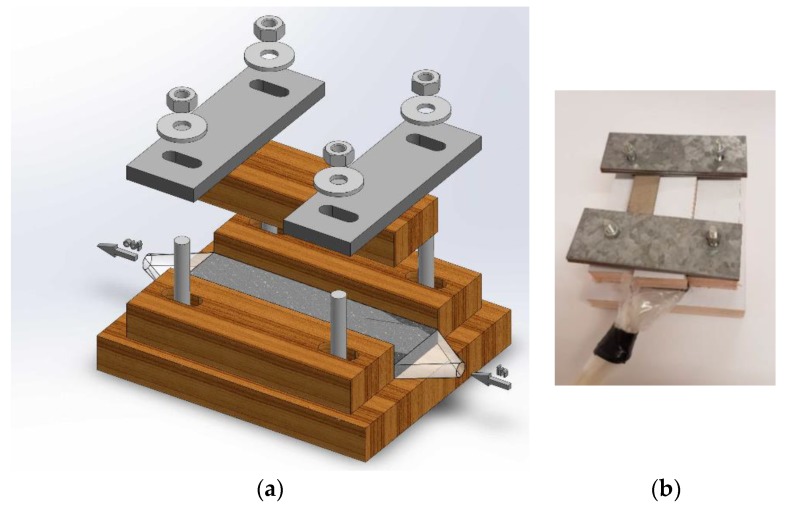
(**a**) 3D drawing and (**b**) a view of experimental cell for hydraulic permeability measurements.

**Figure 3:**
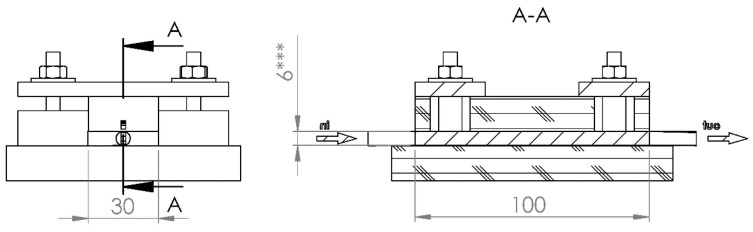
Dimensions of experimental cell.

A special testing cell was made (Figure 2), whose construction permits hydraulic permeability and electric conductivity measurements for differently compressed CF. Cell dimensions (Figure 3) allowed testing felt samples of 47 mm × 28.7 mm, whereas the felt height was reduced by compressing from 6.6 to 1.2 mm using four screw bolts. The symmetry of the felt compression and its height measurements were provided by a digital caliper having 0.01 mm accuracy.

Different flow rates were provided by height adjustment between the water source and the cell. Flow rates were measured with an accurate vessel and stopwatch, while the pressure was measured in meters of water head. Electrical conductivity was measured for the felt located in the cell between two thin copper sheets simulating the electrode’s substrate.

The hydraulic resistance of an individual cell was determined by relying on a Darcy flow regime through a CF. The choice of the Darcy law was due to the proof given in [27,28] showing that the flow rate is linearly proportional to the liquid pressure. Flow value was estimated by the measured volume of a liquid that passed through the experimental cell, divided by the time of passage. Measurements of liquid volume were carried out in a beaker with dimensional divisions of 1 cm^3^. Time was measured by a stopwatch. Each test time was between 2 and 3 min for providing experimental results with an accuracy better than 0.5%. Control and measurements of liquid pressure at the cell entrance were performed by alternating the height of a beaker over the cell. The accuracy of a pressure measurement was not less than 1 cm, which translates to a relative error not worse than 0.1–0.2%. Liquid temperature was evaluated by digital thermometer with an accuracy of 0.1 °C. Due to safety issues, the regime of fluid flow was measured on a water stream across the cell. This is a reliable approach since results from these experiments could be applied to the flow of a real electrolyte, taking into account its dynamic viscosity and density versus the same properties of water. Results of permeability provided an estimation of coefficient *k*/*d*^2^ from Equations (2) and (3).

Electrical conductivity was determined in the same cell for differently compressed CFs, first in dry conditions and later when CF was immersed in three different dielectric liquids having dissimilar electrical permittivity, glycerol, alcohol, and cyclohexane. Conductivity measurement was provided by bench type R/L/C METER IX 3131B able to measure resistance by applying 100 Hz, 120 Hz, 1 kHz, and 10 kHz AC frequencies with an error of ~0.6%. In addition, resistance by applying DC was measured by a multi-meter (Fluke 179 model, Fluke Co., Everett, WA, USA) The results of hydrodynamic investigation and conductivity measurements are presented in the following sections.

### 2.2. Carbon Felt Hydrodynamic Permeability

**Figure 4:**
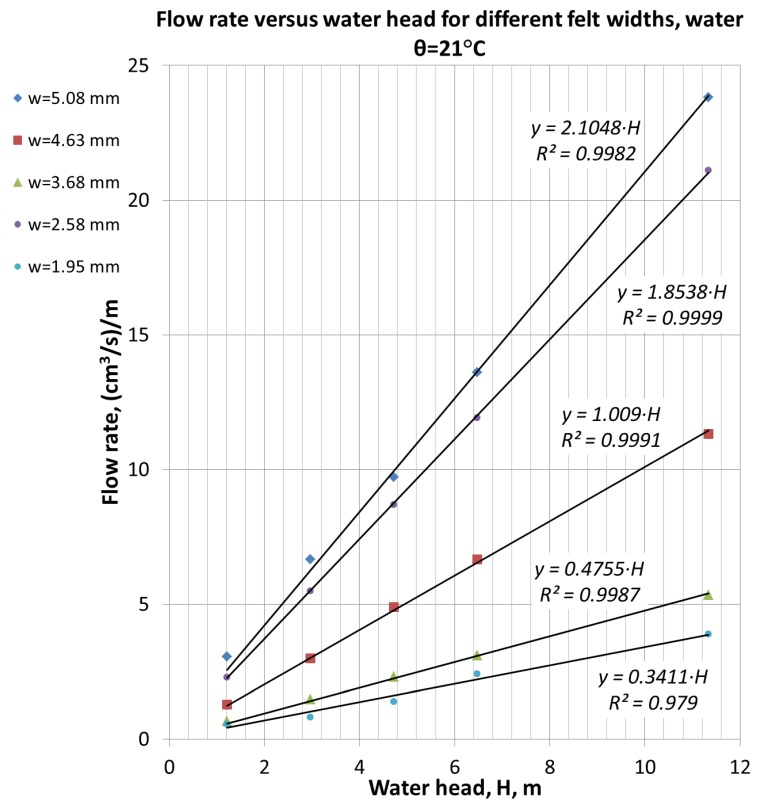
Dependence of a flow rate through cell filled with CF versus water pressure for five different felt thicknesses was obtained by felt compression.

The graphs in Figure 4 present the dependence of flow rate through CF versus water pressure and for differently compressed felts. All given curves have linear dependence and begin from the axis origin, proving the validity of a Darcy flow regime assumption. The initial height of felt is equal to 6.2 mm. The first curve in Figure 3 (*h* = 5.08 mm) was obtained in the slowly compressed felt.

**Figure 5:**
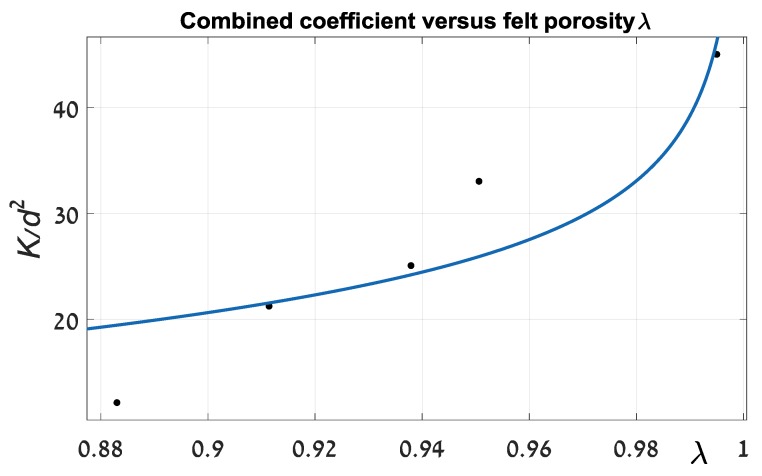
Combined coefficient *k*/*d*^2^ versus felt porosity *λ*.

Although we have shown the linear regime of a liquid flow through CF, it is necessary to understand the slope values. An initial evaluation of coefficient *k*/*d*^2^ (Equations (2) and (3)) was carried out. Below is a graph of its values depending on felt porosity (Figure 5). Approximation was done by special software (MATLAB) fitting procedure.

Magnitudes of coefficients *C* and exponent n from Equation (3) equal 0.07205 (p.u.) and 0.2282 (p.u.), respectively. The proximity criterion (R-squared) is 0.828, which implies a relatively poor convergence between the theoretical and experimental data. Furthermore, the value of exponent *n* is far from that found in the literature. For example, in [16] coefficient n is suggested to be 2. This deficiency significantly limits the practical usefulness of such an approach. The discrepancy in principle could be explained as in Section 1. Theoretical solutions, which are represented in the cited literature sources, are very sensitive to initial assumptions that can be changed by an order of magnitude. It seems that during felt compression, its pattern, which determines the foundation of an analytical solution, is gradually changing. As a result, a strict analytical estimate can differ significantly from real experimental outcomes. For improving the approximation of felt hydraulic resistance, we suggest a more suitable procedure. Firstly, on the basis of experimental data, we established a relationship between average liquid (water, 21 °C) velocities through the felt as a function of a felt width parameter.

The felt width parameter was chosen as its relative decrease from the initial value of uncompressed felt. Average velocity is represented by the coefficient *V_rel_*, which is the velocity in the unit cross section felt area normalized to a unit length and applied over felt one meter pressure head. Knowing *V_rel_* average velocity of a liquid having dynamic viscosity (*µ*, Pa·s) through the felt with length (*L*, cm) and under the pressure head (*H*, m) can be calculated as:

(6) $$V_{av}=V_{rel}\cdot \frac{0.9775\cdot 10^{-3}}{\mu \cdot L}\cdot H,\;cm/s$$

**Figure 6:**
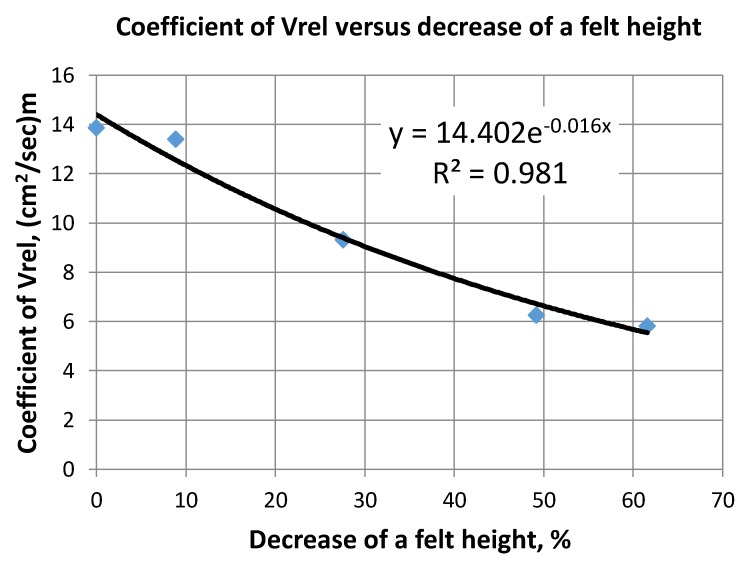
Coefficient of relative velocity through the felt versus decrease of felt width.

Experimentally found values of *V_rel_* (see Figure 6) can be approximated by an exponential function versus felt width decrease *D* (%) as:

(7) $$V_{rel}=14.402\cdot e^{-0.016\cdot D}$$

For CF having an initial carbon content of 4.5–5%, the accuracy of an average liquid velocity assessment (Equation (6)) can be estimated by R^2^ equal to 0.981. This seems to be sufficient for real practical applications.

### 2.3. Electrical Conductivity

**Figure 7:**
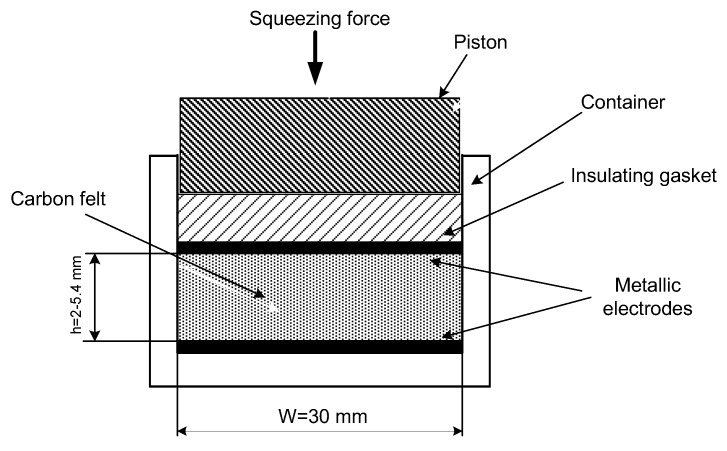
The cell for electrical conductivity measurements.

Electrical conductivity was carried out on the experimental cell (Figure 7) comprising carbon felt between two metallic plates serving as electrodes, insulating gasket and a piston able to compress carbon felt. Carbon felt with electrodes could be placed in different liquids. Electrical measurements were provided by R/L/C METER IX 3131B and their results are presented here both for dry felt electrodes and those immersed in different dielectric (non-conductive) liquids having dissimilar physical parameters (see Table 2).

The reason for using dielectric liquids was to prevent the influence of the (ion) liquid’s inherent conductivity on the resistance of the felt, which was the main object of the investigation. An important assumption regarding the conductivity description was that the resistance of the felt is determined mainly by the density of filament interconnections inside the felt as well as by the density of the filaments’ contacts with electrode surfaces.

Three dissimilar liquids were chosen, glycerol, alcohol, and cyclohexane, all with significantly different electric permittivity ε. Below their most important physical parameters are represented (Table 2).

**Figure 8:**
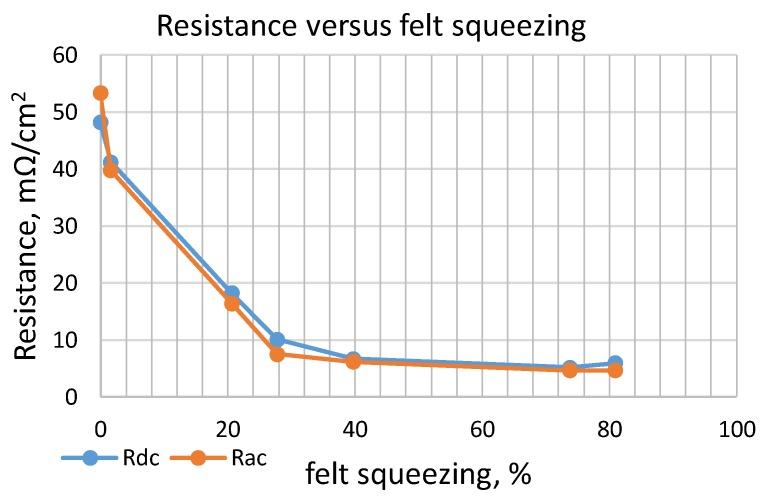
Resistance of a dry felt versus volume compression.

The first set of experiments was conducted with dry CF. Measurement results are shown in Table 3 and Figure 8. Resistance measurements by applying DC or AC current are very similar. Resistivity from the beginning of the carbon compression drops very quickly. Subsequently, its decrease continues much more slowly and after a reduction of 60% of the initial volume (40% of compression), it becomes negligible.

**Figure 9:**
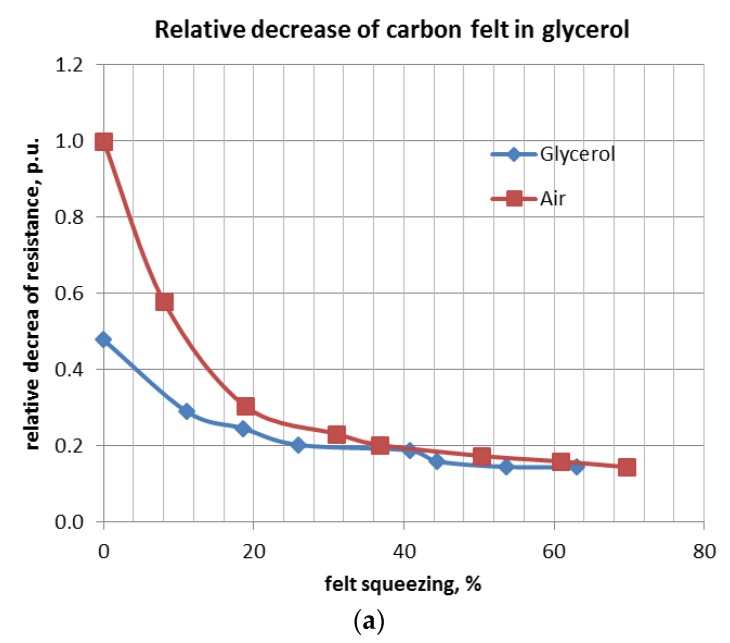

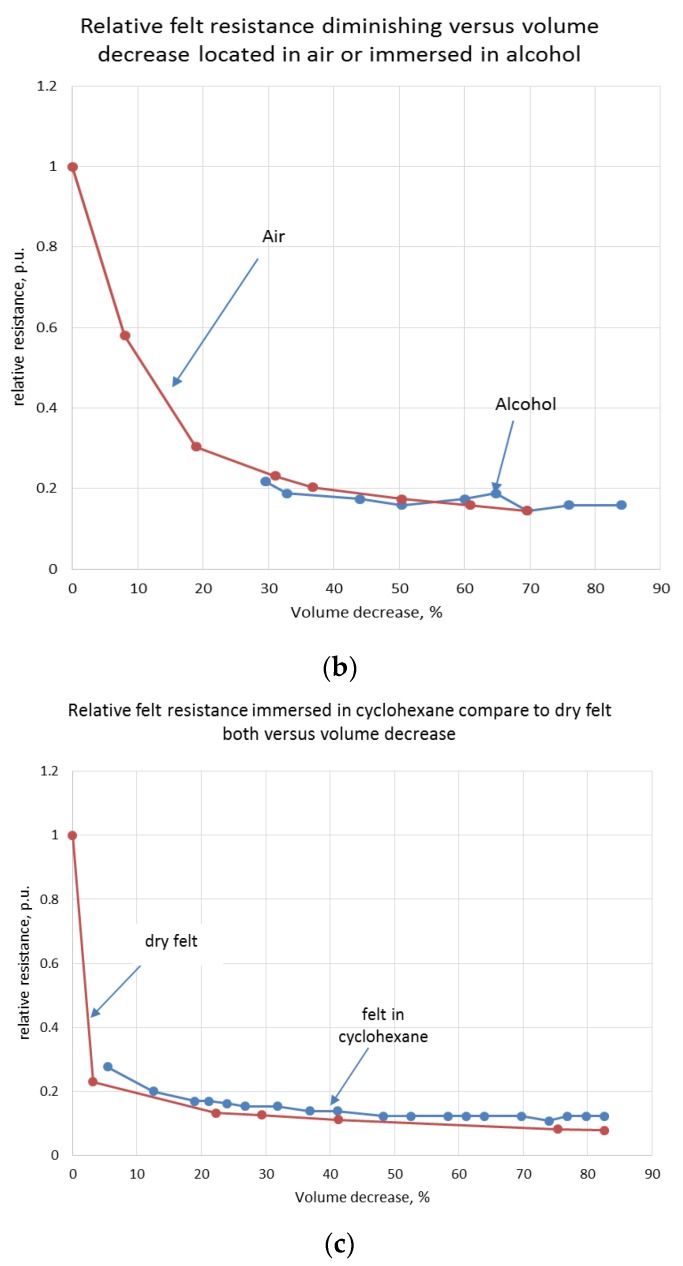
Relative resistance of a felt immersed in glycerol (**a**), alcohol (**b**) and cycloxane (**c**) versus volume compression.

Analogous experiments were carried out for the felt immersed in glycerol, alcohol, and cyclohexane. Curves of relative resistance decrease versus relative felt compression are shown below (Figure 9a–c).

## 3. Conclusions

1. CF material is widely used for electrodes in different electrochemical applications. All of them can be characterized by the presence of electrode reactions in the electrolyte flow. Therefore, the knowledge of electrical and hydraulic properties of CF electrodes has enormous importance for development and exploitation of these electrochemical facilities. New vanadium redox batteries, electrostatic desalination units, and the like could not be optimally designed without such information.
2. The CF framework is represented by a complex pattern of stochastically bundled thin carbon fibers, which to some extent is similar to the porous media, for which pure theoretical approaches have sufficiently low efficiency. Therefore, assessment of hydraulic and electrical CF parameters was carried out in an experimental trial, ensuring good quantitative analytical approximation of obtained results.
3. Hydraulic parameters for each porous medium can be determined by permeability or hydraulic resistivity. Both determine the requirements for pumping equipment and define the pumping specifications for providing electrolyte flow to electrodes. Therefore, they determine the hydraulic efficiency of flow cells with CF electrodes.
4. It should be emphasized that hydraulic resistance (as opposed to permeability) was tested using water due to safety reasons. However, for other liquids (including different electrolyte solutions), the hydraulic resistance or permeability could be recalculated to consider their actual density and viscosity.
5. Hydraulic resistance was found to be linearly proportional to the applied pressure (water head) and drops proportionally to the area of a compressed felt. A special approximating expression for the determination electrolyte flow through differently compression factors of CF was established. This analytical representation can improve the development of further electrochemical applications.
6. The electrical (electronic) conductivity of CF plays a crucial role for electrons to reach the surface of the electrodes and to participate in reactions. Also, electrical resistivity (the inverse of electrical conductivity) plays a significant role in generating electric losses, which are converted to heat, causing temperature problems. In addition, these losses decrease the electrical efficiency of the device.
7. Electronic resistivity was measured by a special device, applying for this purpose DC and AC currents with different frequencies (100 Hz, 120 Hz, 1 kHz, and 10 kHz). Taking into account the requirement to verify the influence of dielectric liquid parameters on CF conductivity, four sets of tests were performed. Resistance was measured in dry conditions, and also with the CF immersed in different non-conducting (dielectric) liquids: glycerol, alcohol, and cyclohexane. Usage of dielectric liquids instead of real electrolytes was justified in order to prevent the influence of electrolyte ionic conductivity on the measurement results.
8. It was observed that electrical resistivity was diminished during felt compression, having a non-linear relationship to volume decrease similar to the negative exponential function. In the initial stage of volume decrease, the resistance drops quickly. However, after 60–80% of its initial value, additional compression has a negligible effect on conductivity.
9. Electrical conductivity moderately depends on liquid permittivity properties. The dielectric constant (ε), among other liquid parameters, obviously has a major influence on the quantity of interconnection between carbon filaments and electrodes. An increase in ε causes a slight improvement in CF conductivity.
10. It seems to be important to continue the present work for finding solid theoretical explanations of all observed experimental data.

## Figures and Tables

**Table 1:** Mechanical properties of CF.

Fiber Diameter, µm	Average	Standard Deviation
	19.2	1.66
Felt density, (kg/m^3^), $Ro_{felt}$	88	-
Carbon density, (kg/m^3^), $Ro_{c}$	1954	-
Porosity, (%), θ	95.5	-
Relative carbon volume, p.u. (%), *V*	0.045 (4.5%)	-
Specific felt surface, (m^−1^), *S*	9.8 × 10^3^	-

**Table 2:** Physical parameters of glycerol, alcohol, and cyclohexane.

Parameter	Liquid		
	Glycerol	Alcohol	Cyclohexane
Density, (g/cm^3^) (25 °C)	1.26	0.789	0.8
Dielectric constant, ε, (p.u.), (0.57 MHz, 25 °C)	~42.5	~21.6	~2.02
Electrical conductivity, ((Ω·cm)^−1^), 25 °C	5 × 10^−8^	~1 × 10^−6^	<5 × 10^−9^
Viscosity, (Pa·s), 20 °C (30 °C)	141 (61.2)	~0.11	0.61

**Table 3:** Resistance of dry CF.

Resistance		h, (mm)						
		6.2	6.1	4.9	4.45	3.7	1.55	1.1
		Volume Decrease, (%)						
		0	2	21	28	40	74	81
Rdc (mΩ·cm^2^)		48.184	41.2	18.2	10.100	6.672	5.189	5.930
Rac, (mΩ/cm^2^)	100, (Hz)	54.855	39.8	16.3	6.820	5.930	4.374	4.374
	120, (Hz)	54.633	38.9	16.6	7.042	6.079	4.522	4.299
	1, (kHz)	54.633	40.1	15.9	6.894	6.375	4.522	4.225
	10, (kHz)	49.296	38.7	14.9	6.746	5.930	4.670	4.299
Rac average, (mΩ·cm^2^)		53.354	39.740	16.38	7.520	6.197	4.655	4.626
